# The potential role of combined shear wave elastography and superb microvascular imaging for early prediction the pathological response to neoadjuvant chemotherapy in breast cancer

**DOI:** 10.3389/fonc.2023.1176141

**Published:** 2023-09-08

**Authors:** Jiaojiao Qi, Chenyu Wang, Yongxin Ma, Jiaxing Wang, Guangfei Yang, Yating Wu, Haiyan Wang, Chengrong Mi

**Affiliations:** ^1^ Department of Obstetrics and Gynecology, General Hospital of Ningxia Medical University, Yinchuan, Ningxia, China; ^2^ Ningxia Medical University, Yinchuan, Ningxia, China; ^3^ Department of Ultrasound, General Hospital of Ningxia Medical University, Yinchuan, Ningxia, China

**Keywords:** breast cancer, neoadjuvant chemotherapy (NAC), superb microvascular imaging (SMI), shear wave elastography (SWE), pathological response

## Abstract

**Objectives:**

The potential role of shear wave elastography (SWE) and superb microvascular imaging (SMI) for early assessment of treatment response to neoadjuvant chemotherapy (NAC) in breast cancer remains unexplored. This study aimed to identify potential factors associated with the pathological response to NAC using these advanced ultrasound techniques.

**Methods:**

Between August 2021 and October 2022, 68 patients with breast cancer undergoing NAC were recruited. Patients underwent conventional ultrasonography, SMI, and SWE examinations at baseline and post-2^nd^ cycle of NAC. Maximum tumor diameter (Dmax), maximum elastic value (Emax), peak systolic velocity (PSV), and resistance index (RI) at baseline and the rate of change of these parameters post-2^nd^ cycle were recorded. After chemotherapy, all patients underwent surgery. Using the Miller-Payne’s grade, patients were categorized into response (grades 3, 4, or 5) and non-response (grades 1 or 2) group. Parameters were compared using t-tests at baseline and post-2^nd^ cycle. Binary logistic regression analysis was used to identify variables and their odds ratios (ORs) related to responses and a prediction model was established. ROC curves were drawn to analyze the efficacy of each parameter and their combined model for early NAC response prediction.

**Results:**

Among the 68 patients, 15(22.06%) were categorized into the non-response group, whereas 53(77.94%) were categorized into the response group. At baseline, no significant differences were observed between the two groups (*p*>0.05). Post-2^nd^ cycle of NAC, rates of change of Emax, PSV and RI (ΔEmax, ΔPSV and ΔRI) were higher in responders than non-responders (*p*<0.05). Binary logistic regression analysis revealed that ΔEmax (OR 0.797 95% CI, 0.683–0.929), ΔPSV (OR 0.926, 95%CI, 0.860–0.998), and ΔRI (OR 0.841, 95%CI, 0.736–0.960) were independently associated with the pathological response of breast cancer after NAC. The combined prediction model exhibited higher accuracy in the early evaluation of the response to NAC (AUC 0.945, 95%CI, 0.873–1.000).

**Conclusion:**

SWE and SMI techniques enable early identification of tumor characteristics associated with the pathological response to NAC and may be potentially indicative of an effective response. These factors may eventually be used for the early assessment of NAC treatment for clinical management.

## 1 Introduction

Breast cancer is one of the leading causes of cancer-related morbidity and mortality in women worldwide (1). Neoadjuvant chemotherapy(NAC) was introduced by Frei in 1982 as a systemic cytotoxic drug treatment for localized tumors prior to radical surgery or radiotherapy (2). According to the current guidelines of the National Comprehensive Cancer Network (NCCN), NAC treatment can not only address locally advanced breast cancer but has also been extended to early operable breast cancer patients (3). However, nearly 20% of patients with NAC may gradually develop drug resistance (4). In instances where the initial phase of NAC treatment is unresponsive or unsatisfactory, the subsequent treatment should be modified accordingly (5). Therefore, early evaluation of the effect of NAC in patients with breast cancer is beneficial for clinical treatment and prognostic evaluation. Pathological assessment is the gold standard for evaluating the efficacy of NAC in patients with breast cancer (6). However, this method is invasive and can lead to a delayed diagnosis. Therefore, imaging techniques play an important role in monitoring the efficacy of NAC in breast cancer.

Currently, several imaging techniques can predict the tumor response to NAC by detecting changes in blood flow and metabolism-related functional indices in the tumor. These techniques include dynamic contrast-enhanced magnetic resonance imaging(MRI), diffusion-weighted imaging, and 18F-FDG PET/CT, which can be used to accurately predict the pathological response of breast cancer to NAC after two cycles of chemotherapy (7, 8). However, MRI and PET examinations are relatively expensive and burdensome for patients undergoing chemotherapy, therefore, their general acceptance among patients is low (9). In contrast, ultrasound is well received by both clinicians and patients because it is safe, noninvasive, and inexpensive. The future development direction lies in integrating multiple ultrasound techniques to assess and predict patient’s responsiveness to NAC therapy in a multidimensional manner, thereby optimizing treatment regimens and avoiding unnecessary toxicity (10).

Angiogenesis promotes tumor growth, and alterations in tumor neovascularization are associated with an impaired chemotherapy response (11). Superb microvascular imaging (SMI) is a new type of ultrasound imaging technology, which can display lower blood flow velocities and smaller blood vessels without using contrast agents (12). Tissue stiffness is another important feature that determines the efficacy of NAC. Shear wave elastography (SWE) tissue elasticity to evaluate the stiffness of the breast lesion, it has the advantages of repeatability and objectivity (13). Existing animal model experimental results have shown that tumor hardness is associated with tumor progression and chemotherapy resistance (14).

Tumor development and infiltration is determined by the tumor microenvironment (15).The SMI technique can indirectly reflect changes in tumor neovascularization, and the SWE technique can indirectly provide insights into the mesenchymal composition of collagen (16).Therefore, the combination of the two advanced techniques is expected to provide a comprehensive prediction of alterations in the tumor microenvironment in patients with breast cancer during NAC treatment, and it holds the potential in sensitively identify patients who are unresponsive to the treatment at an early stage.

SMI combined with SWE can improve the ability to determine the benign/malignant nature of breast cancer (17). However, to the best of our knowledge, few reports have been documented on the use of conventional ultrasound, SWE, and SMI to determine the efficacy of NAC in breast cancer. Therefore, this study explored the potential value of combined SMI and SWE for the early evaluation of the pathological response to NAC, thereby providing a supplementary imaging basis for early clinical assessment.

## 2 Materials and methods

### 2.1 Patients

This retrospective study was conducted from August 2021 to October 2022, during which 68 patients were enrolled. The inclusion criteria were as follows: (i) core needle biopsy confirming the diagnosis of breast cancer; and (ii) clinical decision to treat using NAC. The exclusion criteria were as follows: (i) patients whose lesions were not measurable on imaging; (ii) those who could not tolerate chemotherapy and did not complete the entire cycle of chemotherapy; (iii) those who did not complete the surgery at our hospital; and (iv) those who had incomplete data. This study was approved by the Ethics Committee of the General Hospital of Ningxia Medical University (Ethics No.: KYLL-2022-1090) and written informed consent was obtained from all patients.

### 2.2 US examination

We used an Aplio800 color Doppler ultrasound machine (Canon Medical Systems Corporation, Japan) and an L14-5 high-frequency linear array probe (frequency 5-14 MHz) for examination. All patients underwent conventional ultrasonography, SWE, and SMI before (baseline) and post-2^nd^ cycle of NAC. All ultrasound examinations were performed by a senior ultrasound sonographer with more than 10 years of experience. The Maximum tumor diameter (Dmax) of the tumor was determined from the three grayscale ultrasonography images, and the location of the lesion was marked.

The SMI mode was selected, and the probe was lightly placed on the skin surface, and color gain was adjusted to obtain the maximum blood flow signal. The arterial blood flow pulse was obtained to measure peak systolic velocity (PSV) and resistance index (RI), and the average value was obtained from three measurements. The angle between the ultrasonic sampling line and blood flow direction was maintained below 60°.

**Figure 1:**
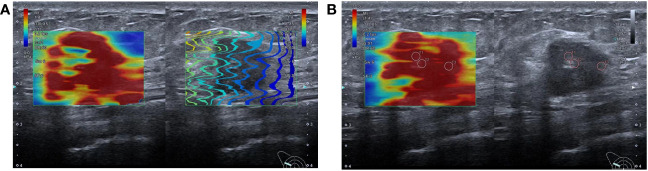
Shear wave elastography schematic diagram: **(A)** SWE imaging quality map, the lines in a rectangular field of view are arranged neatly, indicating that the image quality is good; **(B)** SWE imaging and two-dimensional ultrasound were displayed in split-screen mode. Three ROIs of 2mm were placed at the high-stiffness lesion and the value of mean Emax was 110.0kpa.

SWE was performed in a rectangular field of view, covering the entire lesion and adjacent normal tissues. When the lesion exceeded the detection range, a part of the lesion was selected for measurement. The selected part should exhibit high-quality stiffness that matches the stiffness on the mass map. The quantitative elasticity values in each regions of interest (ROI) (automatically calculated and visualized by the SWE system) were expressed as Young’s modulus (kPa). Three non-overlapping ROI (2 × 2 mm) were placed at the location of lesions with high stiffness, including the stroma around the tumor. The measurements were repeated thrice for each lesion and then averaged (**Figure 1**).

### 2.3 Chemotherapy regimens

Patients (n=68) who participated in this study received 6–8 cycles of preoperative chemotherapy, at an interval of 21 days, and surgery was performed within 2 weeks after the completion of NAC. Chemotherapy regimens strictly followed the NCCN guidelines (3). The detailed treatment protocol was as follows: paclitaxel, epirubicin, and cyclophosphamide in 25 cases; paclitaxel and epirubicin in 15 cases; paclitaxel and carboplatin in 10 cases; and dual-target therapy with docetaxel combined with carboplatin, trastuzumab, and patulizumab in 18 cases.

### 2.4 Pathologic assessments

All pathological sections were scored independently by two pathology teachers under a microscope. All data were obtained after mutual agreement between both parties, each possessing a minimum of 5 years of experience in breast pathological diagnosis. We compared the histopathological findings of tumor lesions isolated during surgery with those of specimens obtained from pre-treatment core needle biopsies to determine the grade of pathological response to NAC in patients with breast cancer. According to the MP classification system (18), we categorized the pathological response to NAC into the following five grades: Grade 1: no significant change in the overall tumor cell density compared to before treatment; Grade 2: continued high overall tumor cell density with a decrease of <30%; Grade 3: tumor cell density decreased by 30%–90%; Grade 4: tumor cell density decreased by >90%; Grade 5: complete disappearance of the tumor, no residual invasive carcinoma detectable under a microscope, though this can include ductal carcinoma in situ. In this study, we designated grades 3–5 as the response group and grades 1–2 as the no-response group based on the difference in pathological response.

### 2.5 Data analysis

At baseline, Dmax, maximum elastic value (Emax), PSV, and RI were expressed as Dmax0, Emax0, PSV0, and RI0, respectively. Post-2^nd^ cycle of NAC, these parameters were expressed as Dmax2, Emax2, PSV2, and RI2, respectively. The differences in the values post-2^nd^ cycle of NAC versus baseline (Δ) were calculated as follows: ΔDmax=100% × (Dmax2-Dmax0)/Dmax0, ΔEmax=100% × (Emax2-Emax0)/Emax0, ΔPSV=100% × (PSV2-PSV0)/PSV0, and ΔRI=100% × (RI2-RI0)/RI0.

### 2.6 Statistical analysis

Statistical analysis was performed using SPSS 26.0 and MedCalc 20.2. Categorical data were expressed as numbers and percentages (%). Measurement data were expressed as mean ± standard deviation. Differences between the response and the non-response groups were assessed using the Student’s t-test, chi-square test, or Fisher’s exact test. For parameters showing statistical significance in the univariate analysis (*p*<0.05), we used binary logistic regression to calculate the odds ratio (OR) and derived a predictive model. Calibration of the model was assessed using the Hosmer–Lemeshow test. The rate of change of each parameter and the value of the model in assessing the pathological response after NAC were also analyzed using receiver characteristic curves (ROC). Areas under the ROC curves (AUC) were compared using the DeLong method to estimate the diagnostic performance of parameters and identify the optimal cut-off value of parameter values for predicting NAC. The optimal cut-off value was calculated by maximizing the Youden index. The performance of the optimal cut-off value for the total points was assessed by sensitivity, specificity, and diagnostic accuracy. AUC >0.9 indicated a good diagnostic value; 0.9 > AUC > 0.7 indicated a moderate diagnostic value; AUC <0.7 indicated a poor diagnostic value (19). A *p* value of <0.05 was considered statistically significant.

## 3 Results

### 3.1 Patient features

The basic clinical and pathological features of the 68 patients included in this study are summarized in **Table 1**. The results of pathological assessment of the postoperative specimens indicated that 53 patients (77.94%, 53/68) were responders to NAC, whereas 15 of them (22.06%, 15/68) were non-responders. We observed 23, 12, 18, 8, and 7 cases of grades V, IV, III, II, and I, respectively. No significant differences were observed in terms of age, menopause, pathological type, histological grade, ER status, PR status, HER2 status, Ki67 index, and lymphatic metastasis at baseline (*p*>0.05).

**Table 1:** Clinical and pathological characteristics of the patients.

Characteristic	Response group(n=53)	Non-response group(n=15)	*p*
Age, y	45.83 ± 11.35	48.20 ± 10.30	0.469
Menopause history Pre−menopause Post−menopause	34(64.7%) 19(35.8%)	10(66.7%) 5(33.3%)	0.857
Pathological type Non-specific invasive carcinoma With others	38(71.7%) 15(28.3%)	12(80%) 3(20%)	0.755
Histologic grades ≤2 >2	49(92.4%) 4(7.6%)	13(86.7%) 2(13.3)	0.856
Estrogen receptor Positive (≥1% IHC) Negative (<1% IHC)	39(73.6%) 14(26.4%)	11(73.3%) 4(26.7%)	1.00
Progesterone receptor Positive (≥1% IHC) Negative (<1% IHC)	35(66.0%) 18(34.0%)	10(66.7%) 5(33.3%)	0.964
HER2 Positive (+3 IHC and/or amplified FISH) Negative (0, + 1 IHC or not amplified FISH)	22(41.5%) 31(58.5%)	6(40.0%) 9(60.0%)	0.916
Ki67 index, n (%)	41.23 ± 37.00	37.00 ± 22.02	0.518
Lymphatic metastasis Positive Negative	43(81.1%) 10(18.9%)	12(80.0%) 3(20.0%)	1.000

Data are presented as mean ± SD and number (percent) where applicable. FISH indicates fluorescence in situ hybridization; and IHC, immunohistochemistry.

### 3.2 Comparison of quantitative parameters between responders and non-responders at baseline and post-2^nd^ cycle of NAC

**Figure 2:**
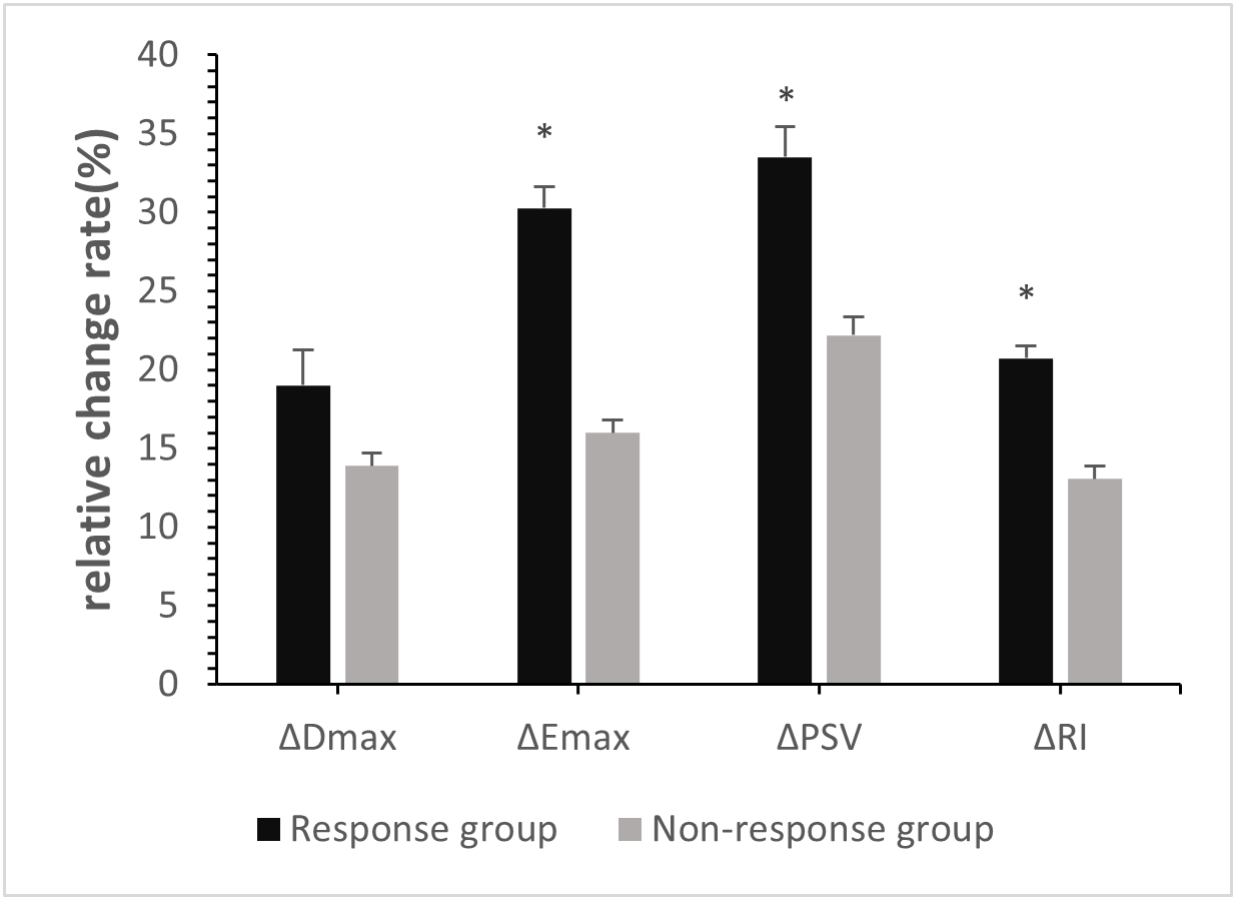
Relative Change rates of quantitative parameters in breast cancer patients post-2^nd^ cycle of NAC. ∗*p <*0:05, compared with non-response group.

**Figure 3:**
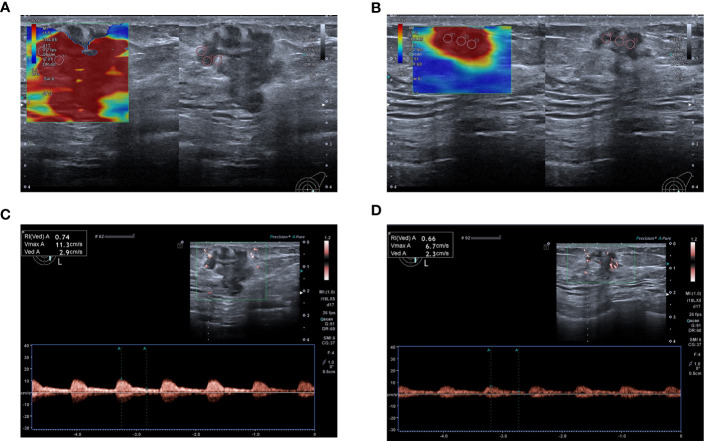
A 59-year-old woman achieved complete response after NAC (Grade 5), and model predictions are greater than the optimal cut-off value. **(A)** Emax measured by SWE before NAC was 108.8 kPa. **(B)** Post- 2^nd^ cycle of NAC, the Emax was 76.9 kPa and the relative change rate of the Emax was 29.32%. **(C)** PSV and RI measured by SMI before chemotherapy were 11.3 cm/s and 0.74 respectively. **(D)** Post-2^nd^ cycle of NAC, the PSV and RI were 4.6cm/s and 0.55 respectively, and the relative change rates of PSV and RI were 59.29% and 25.68% respectively.

At baseline, no significant differences in Dmax, Emax, PSV, or RI were observed between the two groups (*p*>0.05, **Table 2**). Post-2^nd^ cycle of NAC, although no significant difference in the rate of change of Dmax was observed between the two groups (*p*>0.05), the relative rates of change of Emax, PSV, and RI in the response group were significantly higher than those in of the non-response group (*p*<0.05, **Figures 2**, **3**, **Table 3**).

**Table 2:** Comparison of quantitative parameters between response and non-response groups before NAC.

parameters	Response group(n=53)	Non-response group(n=15)	t	*p*
Dmax (cm)	3.10 ± 1.11	3.63 ± 1.19	1.580	0.119
Emax (KPa)	107.69 ± 16.09	106.99 ± 16.88	-0.146	0.884
PSV(cm/s)	21.80 ± 12.28	25.63 ± 14.89	1.107	0.313
RI	0.766 ± 0.045	0.773 ± 0.054	0.498	0.620

Data are presented as mean ± SD where applicable.

**Table 3:** Comparison of quantitative parameters between response group and non-response groups post-2^nd^ cycle of NAC.

Change rate of parameters (%)	Response group(n=53)	Non-response group(n=15)	t	Univariate analysis *p*	logistic regression analysis		
					OR	95%CI	*p*
ΔDmax	-19.02 ± 18.45	-13.90 ± 6.84	1.657	0.103			
ΔEmax	-30.27 ± 10.92	-15.98 ± 6.72	-6.232	0.000*	0.797	0.683-0.929	0.004*
ΔPSV	-33.51 ± 16.10	-22.20 ± 9.69	-3.385	0.002*	0.926	0.860-0.998	0.045*
ΔRI	-20.73 ± 6.34	-13.05 ± 6.64	-4.103	0.001*	0.841	0.736-0.960	0.010*

Data are presented as mean ± SD where applicable. OR, odds ratio; CI, confidence. ^∗^ *p* <0:05.

### 3.3 Factors independently associated with the pathological response of NAC

Among the variables with univariate *p*<0.05, ΔEmax, ΔPSV, and ΔRI were included in the binary logistic regression model. ΔEmax (OR 0.797, 95% CI, 0.683–0.929, *p*=0.004), ΔPSV (OR 0.926, 95% CI, 0.860–0.998, *p*=0.045), and ΔRI (OR 0.841, 95% CI, 0.736–0.960, *p*=0.010) were independently associated with the pathological response of to NAC for in breast cancer (**Table 3**).

### 3.4 The value of independent correlation parameters and combined multiparameter early prediction of pathological response after NAC in patients with breast cancer

**Figure 4:**
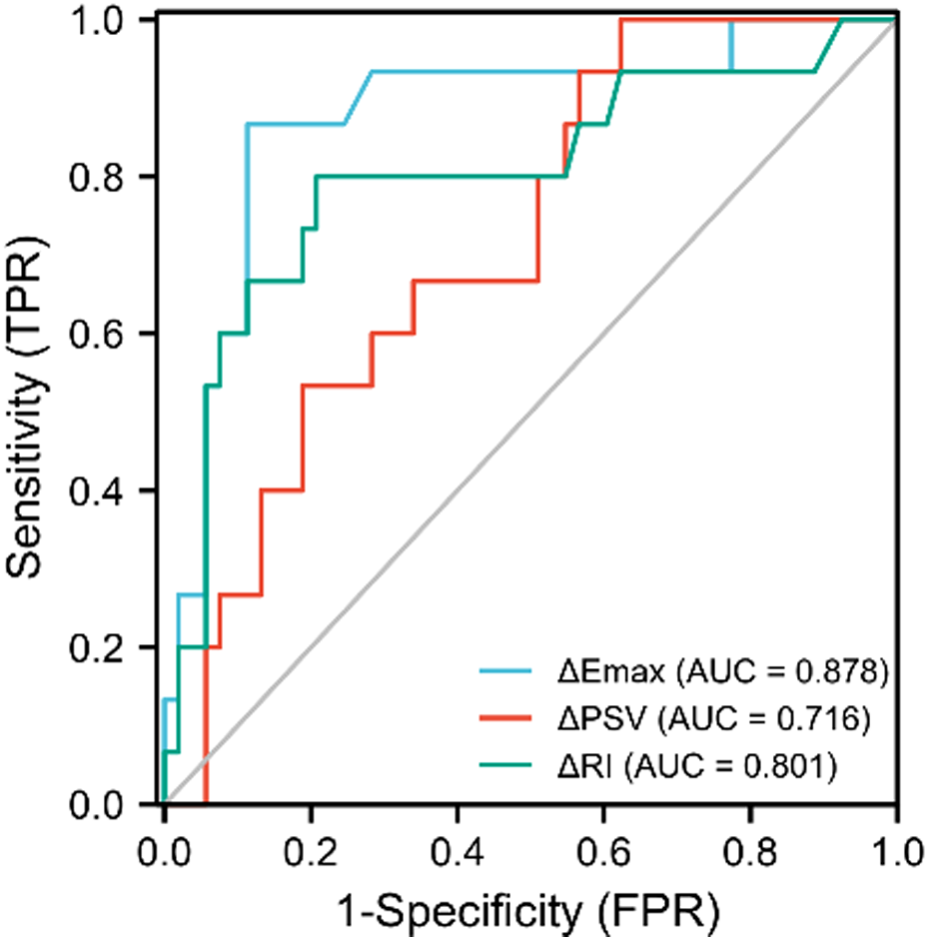
ROC curves of ΔPSV(%), ΔRI(%), and ΔEmax(%) in predicting the efficacy of neoadjuvant chemotherapy for patients with breast cancer.

**Figure 5:**
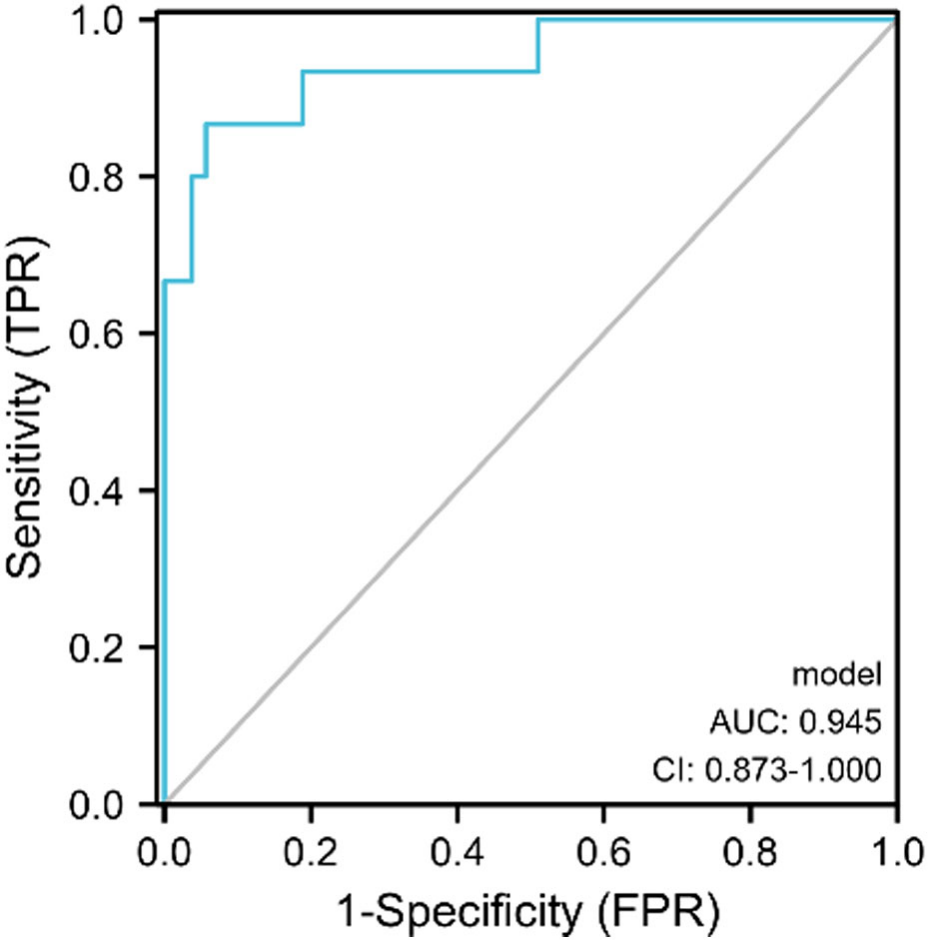
The ROC curve of SMI combined with SWE in predicting the efficacy of NAC for patients with breast cancer.

The ROC curves for ΔEmax, ΔPSV, and ΔRI to distinguish responders, yielded AUCs of 0.878 (CI = 0.769–0.987), 0.716 (CI=0.581–0.850) and 0.801 (CI = 0.654–0.948) with optimal cut-off value were -18.56%, -38.99% and -15.16%, respectively. The Delong test indicated that the combined model encompassing these three parameters for evaluating the efficacy of NAC in patients with breast cancer significantly outperformed the individual factors (*p*<0.05). The AUC value of the combination model was 0.945 (CI = 0.873–1.000), the sensitivity was 94.3%, and the specificity was 86.7%. (**Table 4**, **Figures 4**, **5**).

**Table 4:** SWE and SMI parameters and their combination ROC curve analysis results.

Characters	Cut-off	Sensitivity (%)	Specificity (%)	Accuracy (%)	AUC	95%CI
ΔEmax(%)	-18.56	88.7	86.7	88.2	0.878	0.769-0.987
ΔPSV(%)	-38.99	37.7	100.0	51.5	0.716	0.581-0.850
ΔRI(%)	-15.16	79.2	80.0	79.4	0.801	0.654-0.948
Combination	0.63	94.3	86.7	92.6	0.945	0.873-1.000

We also developed a new prediction model for the pathological response to NAC in patients with breast cancer by combining the SWE and SMI parameter models. The model was obtained by combining ΔEmax, ΔPSV, and ΔRI through binary logistic regression, and the specific equation was as follows: model= − (ΔEmax × 0.228 + ΔPSV × 0.076 + ΔRI × 0.173 + 8.757). The results of the Hosmer–Lemeshow test revealed that the model has *p*>0.10 (0.587), indicating a good fit between the predicted results of the developed model and the actual results.

## 4 Discussion

In clinical practice, ultrasonography plays an important role in evaluating the outcomes of NAC treatment in patients with breast cancer; however, conventional ultrasound alone does not provide sufficient evidence for early assessment. Therefore, acquisition of additional imaging features for evaluating tumor response is becoming an important factor (10), as only a comprehensive assessment of tumor features can precisely evaluate the efficacy of NAC. Moreover, no study has combined SWE and SMI to evaluate the pathological responses to NAC in breast cancer.

In the present study, we used conventional ultrasound, SWE, and SMI techniques to measure the changes in tumor morphology and functionality-related indices after early NAC treatment (2^nd^ cycle), observed that ΔEmax, ΔPSV, and ΔRI were independently correlated with the pathological response of tumors to NAC, and the combined use of these three parameters could more effectively predict the pathological response after NAC than the use of the three parameters alone. This suggested that SWE and SMI hold a great clinical value for the early assessment and prediction of pathological response to NAC in breast cancer. The combined application of these two noninvasive and repeatable ultrasound techniques allows for a comprehensive and timely evaluation of the NAC treatment outcomes.

Currently, Chinese experts strongly recommend that patients with breast cancer receiving NAC should undergo conventional ultrasound at 2-cycle intervals, primarily to monitor changes in the size of breast masses (20). In our study, no significant differences in the maximum diameter of the tumor at baseline and rate of change post-2^nd^ cycle were observed between the two groups. Similarly, Gu’s concluded that a change in tumor diameter after the 1^st^ and 2^nd^ cycles of NAC was not valuable in distinguishing the efficacy of chemotherapy (5). This may be because tumor regression is a slow and gradual process induced by chemotherapy drugs (21). In addition, regression of tumor cells after NAC mainly manifests as necrosis and fibrosis of the lesion (22), and conventional ultrasound cannot accurately differentiate between necrosis, fibroplasia, and residual cancer (23). Therefore, it is impossible to accurately assess tumor regression using conventional ultrasonography.

SWE offers repeatable and quantitative measurement of tumor tissue hardness, not only for early identification of benign and malignant breast lesions but also for monitoring treatment response of the disease (24). Tumor stiffness is closely related to the chemotherapy response in breast cancer. Several studies have confirmed that changes in tumor stiffness serve as early response markers during treatment (24–27). In this study, post-2^nd^ cycle of NAC, the relative rate of change of Emax of tumors in the response group was significantly higher than that in the non-response group (−30.27% vs. −15.98%). Jing et al. reported that the Δ stiffness of responders (−42.19%) was significantly higher than that of non-responders (−23.59%) (27), which is comparable to our results. Both studies acknowledged that responsiveness of patients with breast cancer to NAC is an adaptive process, so the corresponding rate of change in tumor stiffness is more meaningful for predicting efficacy than tumor characteristics acquired at a certain time point. Quantitative SWE parameters were measured in a 3 mm diameter area around the region of interest; however, the region of interest they selected had only one circle, which has the possibility of bias. In our study, we manually placed three circles with a diameter of 2 mm to avoid selection bias.

Contrast-enhanced ultrasonography (CEUS) is superior to conventional imaging methods for measuring angiogenic changes as an indicator of response. Several studies have shown that CEUS can predict the efficacy of NAC for breast cancer after two cycles (28–30); however, the contrast agent used is expensive and has certain restrictions for trauma inspection. SMI is a new and rapidly developing imaging method for evaluating tissue micro-vessels. It has similar ability as CEUS in displaying microvessels and low-speed blood flow within breast lesions (31). Therefore, SMI is expected to serve as a simple, noninvasive, and cost-effective alternative to contrast-based inspection. Li et al. demonstrated the consistency of SMI with histopathology in evaluating the efficacy of NAC in 89 patients with locally advanced breast cancer (107 lesions) (32). Yuan et al. confirmed that the decrease in the post-treatment quantitative parameters, PSV and RI, is related to the pathological response to NAC (33). However, these studies were semi-quantitative, primarily using the Adler flow classification (34), which is susceptible to the operator subjectivity. Therefore, the present study used the rates of change of PSV and RI after two cycles as a quantitative evaluation index. We observed no significant differences in PSV and RI before NAC; however, tumors with higher ΔPSV and ΔRI post-2^nd^ cycle of NAC were more effectively detected. This suggested the potential of ΔPSV and ΔRI in the early assessment and prediction of chemotherapeutic efficacy. Patients with greater rates of change in PSV and RI post-2^nd^ cycle of NAC treatment exhibited a better pathological response to NAC, indicating the efficacy of chemotherapeutic agents to some extent. This could be attributed to the direct action of chemotherapeutic agents in the early stages of NAC, wherein cancer tissue cells and blood vessels that sensitive to chemotherapeutic agents are damaged, resulting in reduced blood flow, a slower blood flow rate, and reduced resistance values (35). This study provides further evidence for early prediction of NAC’s efficacy in patients with breast cancer. Both ΔPSV and ΔRI, especially ΔRI, exhibited high AUC values and could serve as new effective indicators for the early prediction of pathological responses to NACr patients with breast cancer.

The clinical evaluation potential of the advanced ultrasound techniques used in this study suggested that the relative rates of change of Emax, PSV, and RI were effective imaging indicators for early differentiation of pathological responses after NAC, of which ΔEmax was the best, and the combination of the three can could significantly improve the efficacy of NAC (AUC = 0.947). Therefore, combination of SWE and SMI in imaging is advantageous for the early prediction of the pathological response to NAC in BC with breast cancer. Furthermore, our proposed combined prediction model will offer valuable insights for clinical treatment strategies. The amalgamation of the two advanced techniques can comprehensively reflect the changes in the tumor microenvironment of patients with breast cancer during NAC, enabling a sensitive detection of early-stage unresponsiveness. In addition, the ultrasound characterization parameters used in our assessment method are easily measurable and do not require additional intervention. Therefore, SWE combined with SMI is advantageous as a convenient, real-time, cost-effective, and non-invasive imaging approach for the early assessment of NAC efficacy in patients with breast cancer.

This study had some limitations. First, the sample size was relatively small; therefore, tumor heterogeneity and chemotherapeutic regimen were not taken into account. Second, SMI was limited to a specific ultrasound system Canon Aplio. Further studies are required to validate these preliminary findings.

In conclusion, the combination of SWE and SMI may be useful for the early identification of breast cancer response to NAC treatment, with good reproducibility and high sensitivity. In addition, the newly developed predictive model has clinical value for the early prediction of pathological responses after NAC.

## Data availability statement

The original contributions presented in the study are included in the article/supplementary material. Further inquiries can be directed to the corresponding authors.

## Ethics statement

This study was approved by the Ethics Committee of the General Hospital of Ningxia Medical University (Ethics No.: KYLL-2022-1090) and written informed consent was obtained from all patients. Written informed consent to participate in this study was provided by the participants’ legal guardian/next of kin. Written informed consent was obtained from the individual(s), and minor(s)’ legal guardian/next of kin, for the publication of any potentially identifiable images or data included in this article.

## Author contributions

JQ, CM, and CW were responsible for the collection and screening of cases. JQ, YM, JW, GY, and YW performed the statistical analysis and analyzed the data. JQ, YM, and WH wrote and revised the manuscript. WH provided financial support. The final version of the manuscript has been read and approved by all authors, and each author believes that the manuscript represents honest work. All authors contributed to the article.
